# Relationship between paramagnetic rim lesions and slowly expanding lesions in multiple sclerosis

**DOI:** 10.1177/13524585221141964

**Published:** 2022-12-14

**Authors:** Alberto Calvi, Margareta A Clarke, Ferran Prados, Declan Chard, Olga Ciccarelli, Manel Alberich, Deborah Pareto, Marta Rodríguez Barranco, Jaume Sastre-Garriga, Carmen Tur, Alex Rovira, Frederik Barkhof

**Affiliations:** Queen Square MS Centre, Department of Neuroinflammation, Institute of Neurology, Faculty of Brain Sciences, University College London (UCL), London, UK; Section of Neuroradiology, Department of Radiology, Hospital Universitari Vall d’Hebron, Universitat Autònoma de Barcelona, Barcelona, Spain; Queen Square MS Centre, Department of Neuroinflammation, Institute of Neurology, Faculty of Brain Sciences, University College London (UCL), London UK/Centre for Medical Image Computing (CMIC), Department of Medical Physics and Biomedical Engineering, University College London, London, UK/e-Health Centre, Universitat Oberta de Catalunya, Barcelona, Spain; Queen Square MS Centre, Department of Neuroinflammation, Institute of Neurology, Faculty of Brain Sciences, University College London (UCL), London, UK/Biomedical Research Centre, National Institute for Health Research (NIHR) and University College London Hospitals (UCLH), London, UK; Queen Square MS Centre, Department of Neuroinflammation, Institute of Neurology, Faculty of Brain Sciences, University College London (UCL), London, UK/Biomedical Research Centre, National Institute for Health Research (NIHR) and University College London Hospitals (UCLH), London, UK; Section of Neuroradiology, Department of Radiology, Hospital Universitari Vall d’Hebron, Universitat Autònoma de Barcelona, Barcelona, Spain; Section of Neuroradiology, Department of Radiology, Hospital Universitari Vall d’Hebron, Universitat Autònoma de Barcelona, Barcelona, Spain; Neurology-Neuroimmunology Department, Multiple Sclerosis Centre of Catalonia (CEMCAT), Vall d’Hebron Barcelona Hospital Campus, Barcelona, Spain; Neurology-Neuroimmunology Department, Multiple Sclerosis Centre of Catalonia (CEMCAT), Vall d’Hebron Barcelona Hospital Campus, Barcelona, Spain; Queen Square MS Centre, Department of Neuroinflammation, Institute of Neurology, Faculty of Brain Sciences, University College London (UCL), London, UK/Neurology-Neuroimmunology Department, Multiple Sclerosis Centre of Catalonia (CEMCAT), Vall d’Hebron Barcelona Hospital Campus, Barcelona, Spain; Section of Neuroradiology, Department of Radiology, Hospital Universitari Vall d’Hebron, Universitat Autònoma de Barcelona, Barcelona, Spain; Queen Square MS Centre, Department of Neuroinflammation, Institute of Neurology, Faculty of Brain Sciences, University College London (UCL), London, UK/Centre for Medical Image Computing (CMIC), Department of Medical Physics and Biomedical Engineering, University College London, London, UK Biomedical Research Centre, National Institute for Health Research (NIHR) and University College London Hospitals (UCLH), London, UK/Radiology & Nuclear medicine, VU University Medical Centre, Amsterdam, The Netherlands

**Keywords:** Chronic active lesions, paramagnetic rim lesions (PRLs), slowly expanding lesions (SELs), volumetric MRI, susceptibility-weighted imaging (SWI), multiple sclerosis

## Abstract

**Background:**

Magnetic resonance imaging (MRI) markers for chronic active lesions in MS include slowly expanding lesions (SELs) and paramagnetic rim lesions (PRLs).

**Objectives:**

To identify the relationship between SELs and PRLs in MS, and their association with disability.

**Methods:**

61 people with MS (pwMS) followed retrospectively with MRI including baseline susceptibility-weighted imaging, and longitudinal T1 and T2-weighted scans. SELs were computed using deformation field maps; PRLs were visually identified. Mixed-effects models assessed differences in Expanded Disability Status Scale (EDSS) score changes between the group defined by the presence of SELs and or PRLs.

**Results:**

The median follow-up time was 3.2 years. At baseline, out of 1492 lesions, 616 were classified as SELs, and 80 as PRLs. 92% of patients had ⩾ 1 SEL, 56% had ⩾ 1 PRL, while both were found in 51%. SELs compared to non-SELs were more likely to also be PRLs (7% vs. 4%, *p* = 0.027). PRL counts positively correlated with SEL counts (ρ= 0.28, *p* = 0.03). SEL + PRL + patients had greater increases in EDSS over time (beta = 0.15/year, 95% confidence interval (0.04, 0.27), *p* = 0.009) than SEL+PRL-patients.

**Conclusion:**

SELs are more numerous than PRLs in pwMS. Compared with either SELs or PRLs found in isolation, their joint occurrence was associated with greater clinical progression.

## Introduction

Relapse-onset multiple sclerosis (MS) is characterised by the development of acute focal new lesions. While acute inflammation typically lasts weeks to a month, at pathology between 15% and 30% of lesions show evidence of chronic inflammatory activity, with such lesions termed chronic active or smouldering.^1,2^ They show progressive tissue matrix damage in their cores and have rims of iron-laden activated microglia/macrophages with myelin breakdown and reactive astrocytes.^3,4^ Chronic active lesions are seen in all phenotypes of MS and are thought to contribute to clinical progression independent of relapses through neuro-axonal damage.

On magnetic resonance imaging (MRI), the paramagnetic properties of iron-enriched macrophages and microglia at the edge of lesions enable some chronic active lesions to be identified on SWI (termed paramagnetic rim lesions, PRLs). Hypointense rims surrounding lesions have been described in both relapsing and progressive MS using T2*-weighted or phase,^5^ susceptibility-weighted imaging (SWI)^6^ as well as quantitative susceptibility mapping (QSM).^7^ It has also been shown that some PRLs slowly expand over time more so than non-PRLs,^8^ despite other data reporting that shrinkage can also be seen in PRLs.^9^ In MS, PRLs are estimated to occur in about 40% of patients and on average make up ~10% of the overall lesion count.^10^ However, limited MRI sensitivity in detecting intracellular iron^8^ may suggest an underestimation of the true number of such lesions. PRLs are specific for MS^11^ and having four PRLs is associated with greater motor and cognitive disability at an earlier age,^12^ but it remains uncertain if they preferentially accumulate in progressive MS.^13,14^

Chronic lesion activity can also be detected with slowly expanding lesions (SELs) using deformation-based volumetric MRI. As with PRLs, SELs are seen in all MS phenotypes,^15,16^ appear more numerous with progressive (primary and secondary) progressive compared with relapsing-remitting MS,^15^ and have been associated with neurological and cognitive disability.^17,18^ SELs also represent a high fraction of the total lesion burden.^18^ SELs have a greater T1 hypointense volume than non-SELs,^17,19^ lower magnetisation transfer ratio (MTR),^20^ more abnormal diffusivity measures,^21^ and are more likely to become persistent black holes (PBH),^22^ suggesting greater neuro-axonal damage. While both represent chronic active lesions, it is unclear how SELs and PRLs are related, or have different associations with clinical outcomes.

In this work, we hypothesised that (1) lesions with paramagnetic rims would be more likely to expand and so would be classified as SELs; (2) both lesion types would be associated with a higher lesion volume and more abnormal brain-derived measures; and (3) that patients who had both PRLs and SELs would have a poorer clinical prognosis than those who did not.

We aimed to: (1) investigate the association between PRLs, identified on the baseline SWI, and SELs, computed using volumetric MRI, in relapse-onset MS; (2) evaluate their impact on other MS-specific markers (total lesion volume and brain-derived metrics); (3) evaluate groups defined by the presence or absence of PRLs and SELs; (4) assess the independent and combined contribution of PRLs and SELs to the evolution of disability.

## Materials and methods

### Participants, MRI acquisitions and clinical assessments

Participants, who had all given informed consent for use of their data, were retrospectively selected from an observational study conducted at Centre d’Esclerosis Multiple de Catalunya and Section of Neuroradiology, Vall d’Hebron Barcelona Hospital Campus. The study received approval from the local Ethical Commitees of Vall d’Hebron Barcelona Hospital Campus and the Queen Square MS Centre, University College London. The inclusion criteria were: (1) a confirmed diagnosis of relapsing-remitting (RRMS) or clinically isolated syndrome (CIS) according to the revised 2017 McDonald criteria;^23^ (2) availability of baseline SWI and fluid-attenuated inversion recovery (FLAIR) or T2-weighted scans; (3) at least three consecutive 3D magnetisation-prepared rapid gradient-echo (MPRAGE) scans of adequate image quality.

The scans were performed on a 3T system (Tim Trio; Siemens, Erlangen, Germany) using a 12-channel phased-array head coil, with the following acquisition parameters as previously described:^6^ (1) transverse fast FLAIR (TR = 9000 ms, TE = 87 ms, TI = 2500 ms, flip angle = 120°, voxel size = 0.49×0.49×3.0 mm^3^), (2) sagittal T1-weighted 3D MPRAGE (TR = 2300 ms, TE = 2.98 ms, TI = 900 ms, voxel size = 1.0×1.0×1.2 mm^3^) and (3) transverse SWI (TR = 33 ms, TE_1_ = 6.08 ms, TE_2_ = 24.6 ms, flip angle = 15°, voxel size = 0.65×0.65×3 mm).

At each MRI session, Expanded Disability Status Scale (EDSS)^24^ scores were assessed by experienced MS neurologists. Changes in EDSS scores were assessed between baseline and the last MRI session. Confirmed disability progression (CDP) was based on an EDSS change ⩾ 1.5 or ⩾ 1.0 when baseline EDSS score was 0 or > 0, respectively, as previously described,^25^ confirmed 6 months after the last MRI session.

### Lesion and brain segmentation, SEL detection

Lesions were identified using the automated lesion prediction algorithm from the Lesion Segmentation Tool (LST),^26^ part of Statistical Parametric Mapping (SPM) software package, based on the analysis of baseline FLAIR images. The output of the LST segmentation was manually quality-checked and, if needed, corrected using Jim v7.0 (Xinapse Systems, Aldwincle, UK) by experienced raters (AC & MC).

The 2D FLAIR images were resampled to 1 mm isotropic space. The 3D-MPRAGE images were co-registered to the lesion masks.

SEL-derived metrics were obtained with the in-house algorithm:^18^ lesions seen on FLAIR over 10 mm^3^ were classified as SELs or non-SELs based on the presence or absence of detectable expansion (as measured by the Jacobian determinant) on T1-weighted images. To account for differences in the follow-up time intervals, the SEL pipeline incorporated a normalisation step that computed the z-score of Jacobian. In previous work, we subdivided SELs into possible and definite categories, but here in our descriptive analyses we combined both into a single SEL category, also referred to as SEL candidates.^19^ However, the models including the clinical variables were evaluated within all SEL sub-types.

For brain extraction, tissue segmentation and parcellation, Geodesic Information Flows ^27^ was applied to T1-weighted volumetric scans, as previously described.^28^ Using these segmentations, we obtained the normalised brain volume (NBV), normalised cortical grey matter (CGM) and normalised deep grey matter (DGM) volumes.

### PRL detection on SWI

FLAIR and SWI images were rigidly aligned using NiftyReg, and each lesion within the lesion mask was identified with a unique identification number that was kept after transforming the masks to SWI space using nearest neighbour interpolation.^28^ The registered lesions were then assessed by two independent raters (AC and MC), using 3D Slicer, to identify PRLs (on SWI) and confirm the hyperintensity (on FLAIR). The criteria for the definition of a PRL were: (1) a partial or complete rim of hypointense signal relative to the lesion core and surrounding white matter; (2) correspondence to the lesion’s edge on FLAIR; (3) a rim visible on at least two consecutive slices. We excluded PRLs that did not correspond to lesion edges or to hypointense areas on the FLAIR, and care was also taken not to mistake veins and signals from the white/grey matter border for PRLs. The raters independently marked 20 scans before the start of the study (Cohen’s Kappa = 0.87) and discussed why differences had arisen. They then independently reviewed all the remaining cases, and in the case of any disagreements, it was adjudicated by two experienced radiologists (AR and FB).

### Statistical analysis

Analysis was performed with R and statistical significance was reported at *p* < 0.05. Lesions were individually classified as a SEL or a non-SEL, and whether they were also a PRL, allowing the coincidence of SEL and PRL to be assessed at the lesion level. Lesion counts and volumes were also analysed for each subtype at the patient level, reporting median and range, or mean and standard deviation (SD). Lesion volumes were log-transformed to produce more normally distributed values. Associations between measures were initially assessed with Spearman (‘ρ’) correlation coefficients. Then, we performed Poisson and linear regressions, for the counts and the volumes, and checked the normality of the residuals. A Chi-square test was used to assess the differences in the proportion of SEL and non-SEL lesions that were PRLs. Patients were stratified according to the following criteria: ⩾ 1 SEL and ⩾ 1 PRL (SEL + PRL +); ⩾ 1 SEL and 0 PRLs (SEL + PRL−); 0 SELs (SEL−). Since the number of SEL− patients was small, the subsets with and without PRLs were not considered separately. Differences in those subgroups were assessed using linear regressions. The analyses were repeated separating the cohort into those with shorter (< 2 years) and longer (⩾ 2 years) follow-ups.

Mixed-effects regression models adjusted for age, gender, relapse rate, baseline total lesion volume, and treatment status (at the end of the study) were used to assess the relationship between the change in EDSS (dependent variable) and the MRI measures as independent variables, i.e. counts and log-volumes of SELs (possible and definite) as observed over the study period and presence of PRLs at baseline or their co-occurrence. The interaction term between each metric and the time at follow-up was used, and the random effects included the subject identification number. Multiple logistic regressions adjusted for the same covariates were applied to investigate the risk of CDP explained by within-patient counts or log volumes of SELs (possible or definite).

## Results

### Cohort demographics and clinical features

The demographical and clinical characteristics of the cohort are presented in Table 1. The median age at baseline was 34.4 years (range 14.1–64.9); the median disease duration was 0.4 years (range 0.1–16.6) and the majority of patients were female (69%). The median time to the last follow-up MRI scan was 3.2 years (range 0.7–8.3 years). At baseline, 6 patients were classified as having had a CIS and 55 were relapsing-remitting (RRMS); at the last follow-up 6 still had a CIS, 53 were RRMS and 2 converted to secondary-progressive multiple sclerosis (SPMS). The median EDSS at baseline was 1.5 (range 0–4.5), and the mean EDSS change was 0.16 (SD = 1.33). 14 (23%) patients developed CDP by the end of the study. At baseline, 16% of patients were receiving a disease-modifying treatment (DMT), while 84% had been treated with a DMT at the final follow-up.

**Table 1.** Clinical-demographic characteristics of the patients enrolled in the study.

Demographics and clinical features	
Number of patients	61
Female, *n* (% over total)	42 (69 %)
Age at baseline, median (range), years	34.4 (14.1–64.9)
Disease duration, median (range), years	0.4 (0.1–16.6)
Time to intermediate scan, median (range), years	0.8 (0.4–6.4)
Time to final scan, median (range), in years	3.2 (0.7–8.3)
EDSS at baseline, median (range)	1.5 (0–4.5)
EDSS at final scan, median (range)	1.5 (0–5.5)
EDSS change^a^, mean (SD)	0.16 (1.33)
MS phenotype at baseline	CIS = 6; RRMS = 55
MS phenotype at last follow-up	CIS = 6; RRMS = 53; SPMS = 2
Number of relapses between baseline and final scan, median (range)	0 (0–7)
Time elapsed since the last relapse and the baseline scan, median (range), in days	114 (0–2783)
Number (%) of patients with CDP*	14 (23%)
Number (%) of patients treated at baseline	16 (26%)
Number (%) of patients treated at last follow-up Number (%) treated with first line DMT Number (%) treated with first and second line DMTs	51 (84%) 33 (54%) 18 (30%)

EDSS: Expanded Disability Status Scale; SD: standard deviation; MS: multiple sclerosis; CIS: clinically isolated syndrome; RRMS: relapsing-remitting multiple sclerosis; SPMS: secondary-progressive multiple sclerosis; CDP: confirmed disability progression; DMT: disease-modifying treatment.

a EDSS change calculated as the difference between EDSS at final scan and EDSS at baseline.

* CDP was defined by an EDSS change ⩾ 1.5 if baseline EDSS = 0 or EDSS change ⩾ 1.0 when baseline EDSS > 0, respectively, and confirmed at least > 6 months after the last scan.

### The prevalence of SELs and PRLs at a patient level

Nearly all patients had at least one SEL (92%, *n* = 56), while slightly more than half had ⩾ 1 PRL (56%, *n* = 34). Table 2 shows that the majority of patients were SEL + PRL + (*n* = 31, 51%), followed by SEL + PRL− (*n* = 25, 41%) and finally, patients without SELs (SEL−, *n* = 5, 8%). None of the SELs, PRLs or brain volume measures was different between the group followed up for < 2 years (*n* = 15) compared to those followed up for ⩾ 2 years (*n* = 46) (Supplementary Table 1).

**Table 2.** Patient subgroups according to SEL and PRLs.

		Presence of SELs	
		SEL+ (⩾1 SEL) *n* = 56	SEL− *n* = 5
Presence of PRLs	PRL + (⩾ 1 PRL) *n* = 34	31	3
	PRL− *n* = 27	25	2

SEL: slowly expanding lesion; PRL: paramagnetic rim lesion.

### SELs and PRLs, and their co-localisation at a lesion level

**Figure 1.**
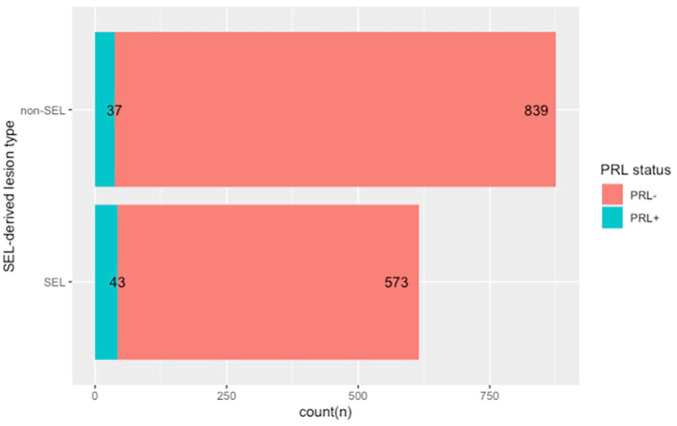
Stacked bar plot showing the total number of lesions classified as SEL or non-SEL, and the correspondent number of PRL within each subtype. The figure, drawn with R, shows the counts of lesions divided between the SEL and the non-SEL categories, and the corresponding PRLs in each subtype. The proportion of PRLs was higher among the SELs than among the non-SELs (7% vs. 4%, *p* = 0.027).

**Figure 2.**
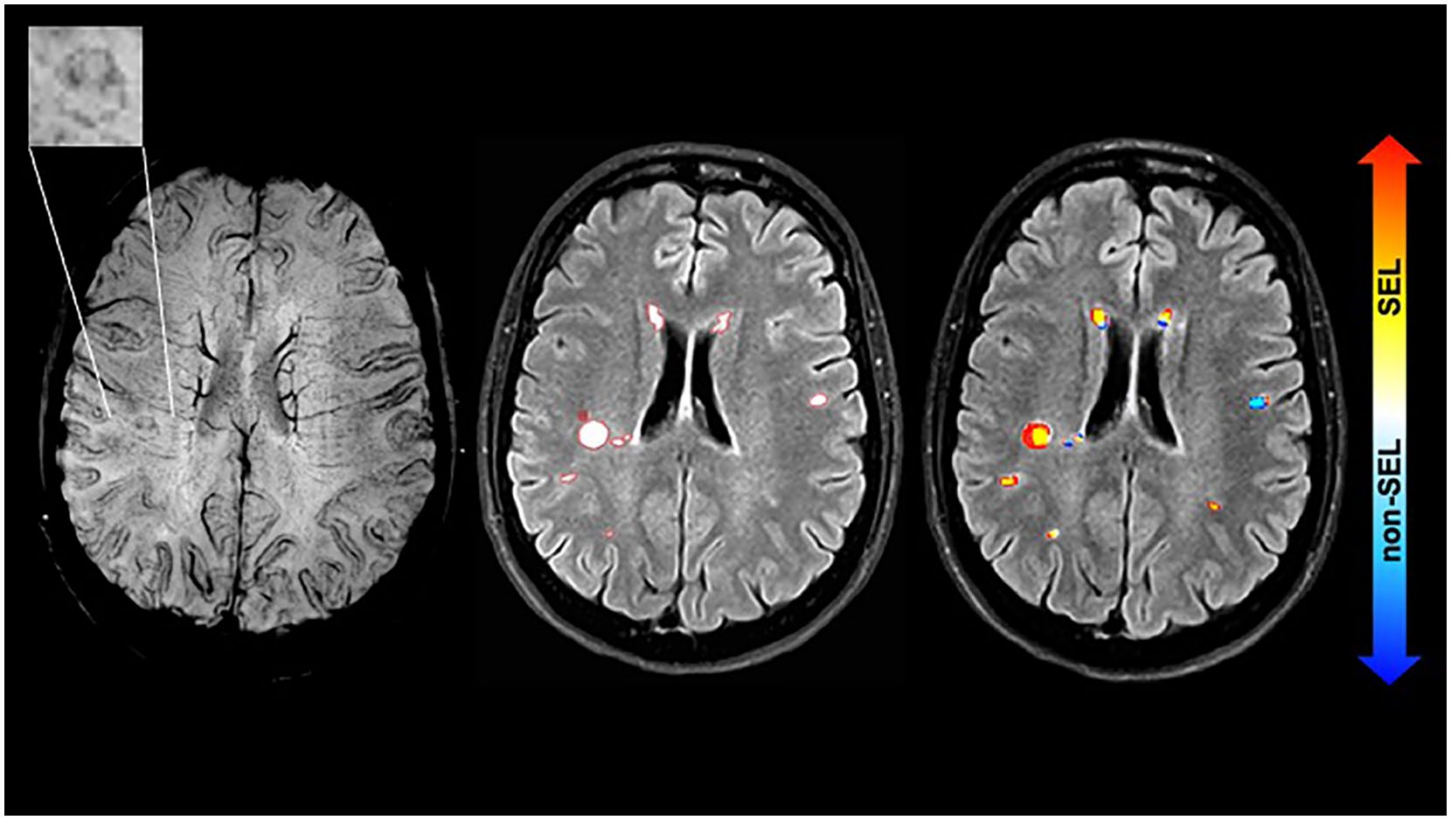
Example of a PRL at baseline and the correspondence to a SEL. The images show from left to right: SWI at baseline (the top left rectangle shows a magnification of the paramagnetic rim lesion), FLAIR with lesion mask countered in red, Jacobian map superimposed indicating that the PRL corresponds to a SEL. The images are from a 45-year-old person with a CIS and the MRI was performed 4 months after onset. At baseline the EDSS was 0 and last follow-up (~4 years) was 1.5. CIS: clinically isolated syndrome; EDSS: Expanded Disability Status Scale; PRL: paramagnetic rim lesion; FLAIR: fluid-attenuated inversion recovery; PRL: paramagnetic rim lesion; SWI: susceptibility-weighted imaging; SEL: slowly expanding lesion.

Overall 1492 lesions were identified. Among those, 616 were SELs (41%) and 876 were non-SEL (59%). On the baseline SWI, 80 PRLs were identified, corresponding to 5% of the total lesions (Table 3). 43 lesions were both SELs and PRLs, representing 54% (43/80) of all the PRLs and 7% (43/616) of all the SELs (Figure 1). The proportion of PRLs was higher among the SELs than among the non-SELs (7% vs. 4%, *p* = 0.027). An example of a lesion that corresponds to both a SEL and a PRL is shown in Figure 2.

**Table 3.** Lesion-level analysis of frequency of SEL-derived lesions by PRL.

Lesion type	PRL	No PRLs	Proportion PRL over lesions of the corresponding type
Total lesions (*n* = 1492)	80	1412	0.05
SEL (*n* = 616)	43	573	0.07
Non-SEL (*n* = 876)	37	839	0.04

PRL: paramagnetic rim lesion; SEL: slowly expanding lesion.

### Associations between SELs, PRLs and brain volume measures

The MRI characteristics of the cohort at the patient level are presented in Table 4. A positive correlation between SEL counts and SEL volumes, with baseline PRL counts was seen (ρ= 0.28, *p* = 0.03 and ρ= 0.29, *p* = 0.02, respectively). However, when we performed a partial correlation between these measures, taking into account the total lesion burden, no significant associations were found. There was a negative association between baseline BPF and SEL volumes (ρ = −0.48, *p* < 0.001), which was accounted for by an association with total lesion volumes in the partial correlations. No significant associations were found between SEL or PRL measures with the regional baseline brain volumes (CGM, DGM).

**Table 4.** Radiological measures: counts and volumes and brain-derived measures.

Radiological measures	
Total lesion count at baseline (n), median [range]	20 [1–80]
SEL count (n), median [range]	5 [0–41]
non-SEL count (n), median [range]	10 [0–55]
PRL count at baseline (n), median [range]	1 [0–8]
PRL/total lesion count ratio, mean (SD)	0.05 (0.10)
SEL/total lesion count ratio, mean (SD)	0.41 (0.23)
Total lesion volume at baseline^a^, median [range], ml	2.2 [0.1–67.0]
Total SEL volume, median [range], ml	0.6 [0–65.3]
Non-SEL volume, median [range], ml	0.9 [0–27.1]
Total SEL/total lesion volume ratio, mean (SD)	0.39 (0.28)
PRL volume at baseline, median [range], ml	0.03 [0–1.0]
PRL/total lesion volume ratio, mean (SD)	0.05 (0.09)
Gadolinium-enhancing lesions at baseline, count (n), median [range]	0 [0–40]
New lesions at final session, count (n), median [range]	1 [0–24]
NBV volume at baseline, mean (SD), ml	1505.7 (75.1)
CGM volume at baseline, mean (SD), ml	839.9 (47.9)
DGM volume at baseline, mean (SD), ml	49.4 (3.7)
BPF at baseline, mean (SD)	0.77 (0.02)

SEL: slowly expanding lesion; PRL: paramagnetic rim lesion; SD: standard deviation; NBV: normalised brain volume; CGM: cortical grey matter; DGM: deep grey matter; BPF: brain parenchymal fraction.

All the measures are referred to the patient level.

a Total lesion volume, and as consequence all the SEL-derived volumes (as subsets of the total volume) were based on the segmentation on the FLAIR image.

### MRI measures in patient subgroups based on the presence of SEL and PRLs

Table 5 presents a descriptive analysis of MRI and clinical measures among patients classified as SEL + PRL +, SEL + PRL− and SEL−. Within the subgroups, SEL + PRL + patients had the highest total lesion counts (median 28, *p* = 0.001) and the highest total lesion volume (median 3.7 ml, *p* = 0.005). Consistent with this, in a multiple linear regression model, the SEL category was associated with total lesion volume (beta = 5.7 ml, 95% confidence interval (CI) (0.8, 10.6), *p* = 0.025), but not baseline NBV or regional brain volumes. The percentages of pwMS who changed from untreated to treated from baseline to the end of the study were not different between the SEL + PRL + and the SEL + PRL− groups (Supplementary Table 2). PwMS who were free from relapses within the 3 months before study entry (*n* = 41) did not reveal significant differences in the MRI measures (e.g. number of SELs and PRLs) compared to those with relapses in the previous 3 months (Supplementary Table 3). In addition, pwMS with ⩾ 5 gadolinium-enhancing lesions had similar MRI characteristics to those with < 5 gadolinium-enhancing lesions (Supplementary Table 4).

**Table 5.** Clinical-demographic and radiological characteristics in groups according to presence of SEL and PRLs.

	SEL + PRL + (*n* = 31)	SEL + PRL− (*n* = 25)	SEL− (*n* = 5)	*p*-value*
EDSS at baseline, median [range]	1.5 [0; 4.5]	1 [0; 3]	1 [0; 2]	0.187^a^
EDSS at final follow-up, median [range]	1.5 [1; 5.5]	1 [0; 5.5]	1 [1; 2]	0.106^a^
EDSS change, mean (SD)	0.27 (1.12)	0.06 (1.62)	0.00 (1.00)	0.806^a^
Age at baseline, median [range]	37.2 [17.7; 67.6]	34.2 [17.5; 47.4]	35.7 [23.5; 41.9]	0.325^a^
Disease duration, median [range]	0.2 [0.1–16.7]	0.4 [0.1–13.5]	0.3 [0.1; 0.3]	0.518^a^
Total lesion count, median [range]	28 [4–80]	9 [1–77]	5 [1–24]	**0.006** ^a^
Non-SEL count, median [range]	15 [1–55]	5 [0–44]	5 [1–24]	**0.029** ^a^
SEL count, median [range]	9 [1–41]	4 [1–39]	0 [0]	**0.008** ^a^
Total lesion volume, median [range]	3.7 [0.5–66.9]	0.7 [0.02–6.0]	0.5 [0.1–27.1]	**0.005** ^a^
Non-SEL volume, median [range]	2.1 [0.1–18.3]	0.4 [0–2.5]	0.5 [0.1–27.1]	**0.020** ^a^
SEL volume, median [range]	1.2 [0.1–65.3]	0.2 [0.01–4.2]	0 [0]	**0.007** ^a^
NBV, mean (SD)	1497.2 (83.4)	1512.4 (64.8)	1525.1 (77.9)	0.636^a^
CGM volume, mean (SD)	834.2 (55.8)	846.5 (38.6)	841.6 (39.6)	0.638^a^
DGM volume, mean (SD)	49.0 (4.2)	49.8 (3.0)	50.5 (3.8)	0.552^a^
BPF, mean (SD)	0.76 (0.02)	0.77 (0.01)	0.77 (0.02)	0.130^a^

SEL: slowly expanding lesion; PRL: paramagnetic rim lesion; EDSS: expanded disability status scale; SD: standard deviation; NBV: normalised brain volume; CGM: cortical grey matter; DGM: deep grey matter; BPF: brain parenchymal fraction.

a Univariate linear regression.

* Bold indicates significant results (*p*-value <0.05).

### The associations of SELs and PRLs with demographics

SEL counts were positively associated with the patients’ age at baseline (ρ= 0.37, *p* = 0.004) as well as the SEL volume (ρ= 0.36, *p* = 0.004). SEL lesion volumes were higher in pwMS with longer disease duration (beta = 0.10, 95% CI (0.07, 0.14), *p* < 0.001) and who were older at baseline (beta = 3.0 95% CI (1.7, 4.3), *p* < 0.001). PRL lesion count and volume were not associated with any of the demographic factors explored. No associations were found between gender and either SEL-derived or PRL measures.

### SEL and PRL associations with EDSS progression

In mixed-effects regression models (see Supplementary Table 5), a greater EDSS increase over time was associated with higher definite SEL counts (beta = 0.01/year, 95% CI (0.001, 0.03), *p* = 0.045) and definite SEL volumes (beta = 0.01/year, 95% CI (0.001, 0.01), *p* = 0.044). When adjusted for age at baseline, sex and disease duration, treatment status, and baseline lesion counts and volumes, the total lesion counts and volumes were also associated with EDSS progression. The presence of ⩾ 1 PRL was associated with a greater increase in EDSS over time (beta = 0.15/year, 95% CI (0.01, 0.30), *p* = 0.044) relative to pwMS without PRLs. The SEL + PRL + subgroup had greater increases in EDSS scores over time than SEL+ PRL− subgroup (SEL + PRL + beta = 0.15/year, 95% CI (0.04, 0.27), *p* = 0.009 vs. SEL+ PRL− beta = −0.02/year 95% CI (−0.13, 0.08), *p* = 0.678). No differences were found between patients in the SEL + PRL + and SEL− subgroups (beta = −0.11/year, 95% CI (−0.44, 0.22), *p* = 0.515) or between SEL + PRL− and SEL− (beta =-0.09/year, 95% CI (−0.42, 0.23) *p* = 0.575). When the model was restricted to the subgroup of subjects with < 5 gadolinium-enhancing lesions, the results did not change. In addition, the exclusion of pwMS who experienced a relapse in the previous 3 months showed similar trends of EDSS increase for the SEL + PRL + groups in comparison to the SEL + PRL− (adjusted difference beta = 0.21, 95% CI (0.01, 0.43), *p* = 0.059). In the logistic model, predicting CDP, SEL and PRL counts and volumes were not statistically significant.

## Discussion

In this work, we assessed the associations between MRI markers of the chronic active lesions in pwMS. We found a moderate association between slowly expanding lesions and SWI rim lesions, and that SELs outnumber PRLs by a factor of 10. In addition, this work suggests that the co-occurrence of both MRI markers is an adverse prognostic factor.

In our MS cohort, we found that 92% developed at least one SEL over a heterogeneous follow-up time (median 3, range 0.7–8.3 years), which was a higher percentage compared to previous studies.^29,16^ However, the proportion of patients with at least one SEL was similarly high in patients with acute lesion activity (89% in Beynon et al.^19^). In our study, the SEL detection rate did not seem to be affected by the presence of patients with high inflammatory activity (*n* = 7 had ⩾ 5 gadolinium-enhancing lesions). Alternative explanations include differences in the SEL detection technique or in the clinical characteristics of the population. On the other hand, 56% had at least one PRLs at SWI, in line with previous reports.^6,12^ Despite being early in their disease course, patients on average had 5 SELs (40% of the overall lesions), comparable to more heterogeneous MS phenotypes (4 to 19.5 SELs per patient).^15,18,30^ By contrast, only a low number of baseline lesions were PRLs (80/1492, 5%), suggesting that close to disease onset only a small subset are characterised by ring-like signal. A recent study evaluating up to 7 years after baseline has shown that PRLs can persist in progressive disease, and a half (50%) of them show a slow volume growth and exhibit more destructive MRI features, as assessed by prolonged T1 relaxation times.^9^ However, it was also suggested that, after a period of growth, PRLs stabilise with reduced susceptibility and rim attenuation thereafter, and eventually some lose their rims.

This work indicates a moderate association between PRLs and SELs in recently diagnosed relapse-onset MS. Despite this, the proportion of PRLs that were also classified as SELs exceeded the proportion of PRLs classified as non-SELs. Moreover, the group of patients having both SELs and PRLs (SEL+ PRL+) had the highest lesion counts and volumes. On 7T MRI, an association between lesion expansion and PRLs was previously found.^8^ Newly developed PRLs have been shown to expand by ~30% in volume over 3 years,^8^ while after 7–10 years on average 50% of PRLs expand by > 10% of their initial volume.^9^ Despite the PRLs and SELs being associated in our work, their correlation appears to be mainly driven by the total lesion load, consistent with previous observations.^9,12^

This study found associations of lower BPF with higher SEL volumes, suggesting a potential link between chronic inflammation and global neuro-axonal damage. However, no clear differences in brain volume measures were found between the SEL+ PRL+ and other subgroups, which may be due to the short disease duration of the present cohort.

From a clinical perspective, our results suggest that there is an association between SELs, higher age at baseline and longer disease duration, which are well-known predictors for a worse clinical outcome in MS.^31,32^ The presence of SEL and PRLs in combination predicted clinical disability, as measured by the EDSS, suggesting that as additive factors they might be involved in the perpetuation of inflammation and reduced remyelination. The early deposition of paramagnetic materials within PRLs could indicate a specific adverse pathogenetic process, such as a more aggressive macrophages-microglia profile,^33^ favouring expansion and conversion to SEL.

This work has several limitations. First of all, the MRI scans were retrospectively collected, thus increasing the heterogeneity of the cohort. In addition, the acquisitions of the SWI images were in 2D, which could impact the visualisation of PRLs. However, the use of 3D T1-weighted images for the SEL computation ensured a superior spatial resolution. Second, the short period of observation for this cohort could have impacted the accuracy of SEL detection. Third, the criteria for SEL definition and pipeline used differ between centres, thus making comparisons across studies difficult. Due to the high prevalence of SELs within the cohort, an assessment of patients based on the presence or absence of SELs alone could be of limited clinical value. From the distribution of the SEL counts observed, a cut-off of four SELs may be worth considering in future studies. This study was also limited by having a baseline SWI scan only and the temporal evolution of PRLs could not be assessed or compared with SELs. Other studies, however, have not found a large change in the proportion of PRL over time.^9^ The relationship between contrast enhancement, SELs and PRLs, was not assessed. However, when we excluded the group of pwMS with an outlier gadolinium lesion count or relapse activity in the last 3 months, the results of the models assessed remained materially unchanged. As a recently diagnosed MS cohort, they had a limited disability progression, and only a small minority converted to a secondary-progressive form. As a result, the models assessing disability had small effect sizes or were borderline significant. Finally, assessing the effects of treatment exposure was not an objective of the study, due to the relatively small sample size. However, the percentages of patients who changed treatment over the course were similar and the treatment status at the end of the study was not significantly associated with the EDSS, which suggests that for our sample treatment was unlikely to have had a substantial effect on the group differences observed.

In conclusion, this study suggests that SELs are a common finding and a subset of them can coincide with PRLs. Despite a modest association of PRLs and SELs, their combination, in association with an overall increase in lesion burden, could be of prognostic relevance as a predictor of MS severity early in the disease course.

## Supplemental Material

sj-docx-1-msj-10.1177_13524585221141964 – Supplemental material for Relationship between paramagnetic rim lesions and slowly expanding lesions in multiple sclerosis Supplemental material, sj-docx-1-msj-10.1177_13524585221141964 for Relationship between paramagnetic rim lesions and slowly expanding lesions in multiple sclerosis by Alberto Calvi, Margareta A Clarke, Ferran Prados, Declan Chard, Olga Ciccarelli, Manel Alberich, Deborah Pareto, Marta Rodríguez Barranco, Jaume Sastre-Garriga, Carmen Tur, Alex Rovira and Frederik Barkhof in Multiple Sclerosis Journal

## References

[1] Luchetti S Fransen NL van Eden CG , et al. Progressive multiple sclerosis patients show substantial lesion activity that correlates with clinical disease severity and sex: A retrospective autopsy cohort analysis. Acta Neuropathol 2018; 135(4): 511–528. 29441412 10.1007/s00401-018-1818-y PMC5978927

[2] Frischer JM Weigand SD Guo Y , et al. Clinical and pathological insights into the dynamic nature of the white matter multiple sclerosis plaque. Ann Neurol 2015; 78(5): 710–721. 26239536 10.1002/ana.24497 PMC4623970

[3] Bagnato F Hametner S Yao B , et al. Tracking iron in multiple sclerosis: A combined imaging and histopathological study at 7 tesla. Brain 2011; 134(Pt. 12): 3602–3615. 22171355 10.1093/brain/awr278 PMC3235560

[4] Popescu BF Frischer JM Webb SM , et al. Pathogenic implications of distinct patterns of iron and zinc in chronic MS lesions. Acta Neuropathol 2017; 134(1): 45–64. 28332093 10.1007/s00401-017-1696-8 PMC5486634

[5] Yao B Ikonomidou VN Cantor FK , et al. Heterogeneity of multiple sclerosis white matter lesions detected with T2*-weighted imaging at 7.0 tesla. J Neuroimaging 2015; 25(5): 799–806. 25657078 10.1111/jon.12193 PMC5613291

[6] Clarke MA Pareto D Pessini-Ferreira L , et al. Value of 3T susceptibility-weighted imaging in the diagnosis of multiple sclerosis. AJNR Am J Neuroradiol 2020; 41(6): 1001–1008. 32439639 10.3174/ajnr.A6547 PMC7342768

[7] Zhang Y Gauthier SA Gupta A , et al. Quantitative susceptibility mapping and R2* measured changes during white matter lesion development in multiple sclerosis: Myelin breakdown, myelin debris degradation and removal, and iron accumulation. AJNR Am J Neuroradiol 2016; 37(9): 1629–1635. 27256856 10.3174/ajnr.A4825 PMC5018433

[8] Dal-Bianco A Grabner G Kronnerwetter C , et al. Slow expansion of multiple sclerosis iron rim lesions: Pathology and 7 T magnetic resonance imaging. Acta Neuropathol 2017; 133(1): 25–42. 27796537 10.1007/s00401-016-1636-z PMC5209400

[9] Dal-Bianco A Grabner G Kronnerwetter C , et al. Long-term evolution of multiple sclerosis iron rim lesions in 7 T MRI. Brain 2021; 144(3): 833–847. 33484118 10.1093/brain/awaa436

[10] Ng Kee Kwong KC Mollison D Meijboom R , et al. The prevalence of paramagnetic rim lesions in multiple sclerosis: A systematic review and meta-analysis. PLoS ONE 2021; 16: e0256845. 10.1371/journal.pone.0256845 PMC8425533 34495999

[11] Meaton I Altokhis A Allen CM , et al. Paramagnetic rims are a promising diagnostic imaging biomarker in multiple sclerosis. Mult Scler J. Epub ahead of print 26 August 2022. DOI: 10.1177/13524585221118677. PMC9679799 36017870

[12] Absinta M Sati P Masuzzo F , et al. Association of chronic active multiple sclerosis lesions with disability in vivo. JAMA Neurol 2019; 76: 1474–1483. 31403674 10.1001/jamaneurol.2019.2399 PMC6692692

[13] Mehta V Pei W Yang G , et al. Iron is a sensitive biomarker for inflammation in multiple sclerosis lesions. PLoS ONE 2013; 8(3): e57573. 10.1371/journal.pone.0057573 PMC3597727 23516409

[14] Harrison DM Li X Liu H , et al. Lesion heterogeneity on high-field susceptibility MRI is associated with multiple sclerosis severity. AJNR Am J Neuroradiol 2016; 37(8): 1447–1453. 26939635 10.3174/ajnr.A4726 PMC4983536

[15] Elliott C Wolinsky JS Hauser SL , et al. Slowly expanding/evolving lesions as a magnetic resonance imaging marker of chronic active multiple sclerosis lesions. Mult Scler 2019; 25(14): 1915–1925. 30566027 10.1177/1352458518814117 PMC6876256

[16] Preziosa P Pagani E Meani A , et al. Slowly expanding lesions predict 9-year multiple sclerosis disease progression. Neurol Neuroimmunol Neuroinflamm 2022; 9(2): e1139. 10.1212/NXI.0000000000001139 PMC8808355 35105685

[17] Elliott C Belachew S Wolinsky JS , et al. Chronic white matter lesion activity predicts clinical progression in primary progressive multiple sclerosis. Brain 2019; 142(9): 2787–2799. 31497864 10.1093/brain/awz212 PMC6736181

[18] Calvi A Carrasco FP Tur C , et al. Association of slowly expanding lesions on MRI with disability in people with secondary progressive multiple sclerosis. Neurology 2022; 98(17): e1783–e1793. 10.1212/WNL.0000000000200144 35277438

[19] Beynon V George IC Elliott C , et al. Chronic lesion activity and disability progression in secondary progressive multiple sclerosis. BMJ Neurol Open 2022; 4(1): e000240. 10.1136/bmjno-2021-000240 PMC9185385 35720980

[20] Elliott C Belachew S Fisher E , et al. MRI characteristics of chronic MS lesions by phase rim detection and/or slowly expanding properties (4101). Neurology 2021; 96(15 Suppl.): 4101.

[21] Klistorner S Barnett MH Yiannikas C , et al. Expansion of chronic lesions is linked to disease progression in relapsing–remitting multiple sclerosis patients. Mult Scler 2021; 27(10): 1533–1542. 33215557 10.1177/1352458520974357

[22] Calvi A Tur C Chard D , et al. Slowly expanding lesions relate to persisting black-holes and clinical outcomes in relapse-onset multiple sclerosis. Neuroimage Clin 2022; 35: 103048. 35598462 10.1016/j.nicl.2022.103048 PMC9130104

[23] Thompson AJ Banwell BL Barkhof F , et al. Diagnosis of multiple sclerosis: 2017 revisions of the McDonald criteria. Lancet Neurol 2018; 17(2): 162–173. 29275977 10.1016/S1474-4422(17)30470-2

[24] Kurtzke JF . Rating neurologic impairment in multiple sclerosis: An expanded disability status scale (EDSS). Neurology 1983; 33(11): 1444–1452. 6685237 10.1212/wnl.33.11.1444

[25] Coles AJ Twyman CL Arnold DL , et al. Alemtuzumab for patients with relapsing multiple sclerosis after disease-modifying therapy: A randomised controlled phase 3 trial. Lancet 2012; 380(9856): 1829–1839. 23122650 10.1016/S0140-6736(12)61768-1

[26] Roura E Oliver A Cabezas M , et al. A toolbox for multiple sclerosis lesion segmentation. Neuroradiology 2015; 57(10): 1031–1043. 26227167 10.1007/s00234-015-1552-2

[27] Cardoso MJ Modat M Wolz R , et al. Geodesic information flows: Spatially-variant graphs and their application to segmentation and fusion. IEEE Trans Med Imaging 2015; 34(9): 1976–1988. 25879909 10.1109/TMI.2015.2418298

[28] Prados F Cardoso MJ Kanber B , et al. A multi-time-point modality-agnostic patch-based method for lesion filling in multiple sclerosis. Neuroimage 2016; 139: 376–384. 27377222 10.1016/j.neuroimage.2016.06.053 PMC4988790

[29] Elliott C Arnold DL Chen H , et al. Patterning chronic active demyelination in slowly expanding/evolving white matter MS lesions. AJNR Am J Neuroradiol 2020; 41(9): 1584–1591. 32819894 10.3174/ajnr.A6742 PMC7583098

[30] Elliott C Arnold DL Chen H , et al. Patterning chronic active demyelination in slowly expanding/evolving white matter MS lesions. AJNR Am J Neuroradiol 2020; 41(9): 1584–1591. 32819894 10.3174/ajnr.A6742 PMC7583098

[31] University of California San Francisco MS-EPIC Team: Cree BA Gourraud PA Oksenberg JR , et al. Long-term evolution of multiple sclerosis disability in the treatment era. Ann Neurol 2016; 80(4): 499–510. 27464262 10.1002/ana.24747 PMC5105678

[32] Dekker I Eijlers AJC Popescu V , et al. Predicting clinical progression in multiple sclerosis after 6 and 12 years. Eur J Neurol 2019; 26(6): 893–902. 30629788 10.1111/ene.13904 PMC6590122

[33] Zarruk JG Berard JL Passos dos Santos R , et al. Expression of iron homeostasis proteins in the spinal cord in experimental autoimmune encephalomyelitis and their implications for iron accumulation. Neurobiol Dis 2015; 81: 93–107. 25724358 10.1016/j.nbd.2015.02.001

