# Supplementary material for: Relationship between paramagnetic rim lesions and slowly expanding lesions in multiple sclerosis
Source: Mult Scler. 2022 Dec 14;29(3):352–62. doi: 10.1177/13524585221141964 (PMC9972234; doi:10.1177/13524585221141964)
Supplement: sj-docx-1-msj-10.1177_13524585221141964 – Supplemental material for Relationship between paramagnetic rim lesions and slowly expanding lesions in multiple sclerosis [file sj-docx-1-msj-10.1177_13524585221141964.docx]

**Supplementary Materials**

**Supplementary Table 1**. SEL- and PRL-derived metrics at the patient level in the short- and long-time range interval sub-cohorts

|  | **Patients followed-up for < 2 years**  **[n=15]** | **Patients followed-up for ≥ 2 years**  **[n=46]** | **p value*** |
| --- | --- | --- | --- |
| Total lesions count at baseline (n), median [range] | 18  [2 – 80] | 20  [1 – 77] | 0.750 |
| SEL count at baseline (n), median [range] | 5  [0 – 41] | 5  [0 – 41] | 0.960 |
| Non-SEL count at baseline (n), median [range] | 16  [1 – 55] | 9  [0 – 50] | 0.580 |
| PRL count at baseline (n), median [range] | 1  [0 – 6] | 1  [0 – 8] | 0.750 |
| Total lesion volume at baseline (ml), median [range] | 2.3  [0.1 – 9.7] | 2.1  [0.1 – 66.9] | 0.624 |
| SEL volume at baseline (ml), median [range] | 0.6  [0 – 3.8] | 0.6  [0 – 65.3] | 0.496 |
| Non-SEL volume at baseline (ml), median [range] | 1.6  [0.1 – 6.4] | 0.9  [0 – 27.1] | 0.863 |
| PRL volume at baseline (ml), median [range] | 0.1  [0 – 0.8] | 0.1  [0 – 1.0] | 0.828 |

*unpaired sample t-test (for the normally distributed variables) or Mann-Whitney test (for the non-normally distributed variables)

**Supplementary Table 2.** Treatment status in the patient categories according to SELs and PRLs combinations

|  | **All patients**  **(n=61)** | **SEL+PRL+**  **(n=31)** | **SEL+PRL-**  **(n=25)** | **SEL-**  **(n=5)** |
| --- | --- | --- | --- | --- |
| **Treated at baseline, n (%)** | 16 (26%) | 13 (42%) | 3 (12%) | 0 (0%) |
| **Treated at final scan, n (%)** | 51 (84%) | 30 (97%) | 16 (64%) | 5 (100%) |
| **Change from untreated to treated from baseline to final scan** | 35 (57%) | 17 (55%) | 13 (52%) | 5 (100%) |

The percentage of patients who changed from untreated to treated by the end of the study was not significantly different between SEL+PRL+ and SEL+PRL- groups (p>0.05).

**Supplementary Table 3.** SEL and PRL-derived metrics at the patient level in the group with or without a history of relapses ≤3 months before study entry.

|  | **Patients without relapses within 3 months before study entry**  **[n=41]** | **Patients with relapses within 3 months before study entry [n=20]** | **p value*** |
| --- | --- | --- | --- |
| Total lesions count at baseline (n), median [range] | 15  [1 – 80] | 22  [3 – 66] | 0.564 |
| SEL count at baseline (n), median [range] | 7  [0 – 41] | 4.5  [0 – 39] | 0.410 |
| Non-SEL count at baseline (n), median [range] | 7  [0 – 55] | 16  [2 – 39] | 0.183 |
| PRL count at baseline (n), median [range] | 1  [0 – 8] | 1  [0 – 6] | 0.565 |
| Total lesion volume at baseline (ml), median [range] | 2.0  [0.1 – 66.9] | 2.6  [0.2 – 27.1] | 0.783 |
| SEL volume at baseline (ml), median [range] | 0.7  [0 – 65.3] | 0.4  [0 – 3.3] | 0.535 |
| Non-SEL volume at baseline (ml), median [range] | 0.7  [0 – 13.9] | 1.7  [0.1 – 27.1] | 0.332 |
| PRL volume at baseline (ml), median [range] | 0.01  [0 – 1.0] | 0.03  [0 – 1.0] | 0.802 |
| BPF median (range) | 0.77  [0.72 – 0.79] | 0.76  [0.74 – 0.79] | 0.712 |

*unpaired sample t-test (for the normally distributed variables) or Mann-Whitney test (for the non-normally distributed variables)

**Abbreviations**: SEL= slowly expanding lesion; BPF = brain parenchymal fraction

**Supplementary Table 4.** SEL and PRL-derived metrics at the patient level in the group with ≥5 or <5 gadolinium-enhancing lesions at study onset.

|  | **Patients with <5 gadolinium-enhancing lesions**  **[n=54]** | **Patients with ≥5 gadolinium-enhancing lesions [n=7]** | **p value*** |
| --- | --- | --- | --- |
| Total lesions count at baseline (n), median [range] | 14.5 [1 – 80] | 28 [21 – 54] | 0.083 |
| SEL count at baseline (n), median [range] | 5 [0 – 41] | 8 [0 – 23] | 0.734 |
| Non-SEL count at baseline (n), median [range] | 7 [0 – 55] | 23 [15 – 39] | 0.011 |
| PRL count at baseline (n), median [range] | 1 [0 – 8] | 1 [0 – 6] | 0.543 |
| Total lesion volume at baseline (ml), median [range] | 1.80 [0.02 – 66.93] | 3.25 [1.27 – 27.16] | 0.066 |
| SEL volume at baseline (ml), median [range] | 0.49 [0 – 65.34] | 0.68 [0 – 2.51] | 0.767 |
| Non-SEL volume at baseline (ml), median [range] | 0.84 [0 – 13.94] | 2.44 [0.90 – 27.14] | 0.013 |
| PRL volume at baseline (ml), median [range] | 0.03 [0 – 0.99] | 0.04 [0 – 1.00] | 0.650 |
| BPF median (range) | 0.77 [0.72 – 0.79] | 0.76 [0.74 – 0.79] | 0.839 |

*unpaired sample t-test (for the normally distributed variables) or Mann-Whitney test (for the non-normally distributed variables)

**Abbreviations**: SEL= slowly expanding lesion; BPF = brain parenchymal fraction

**Supplementary Table 5.** Association between the SEL-PRL measures and categories with EDSS over time using mixed-effects regression models

|  | **MRI and patient category**  **(Independent variables)** | **EDSS**  **(Dependent variable)** | |
| --- | --- | --- | --- |
| **Model** | **Interaction terms with time** | **Beta coefficient [95% CI]** | **p value** |
| **1** | **definite SEL count (n)** | beta=0.01/year  [0.001; 0.03] | **p=0.045** |
| **2** | **definite SEL volume* (ml)** | beta=0.01/year  [0.001; 0.01] | **p=0.044** |
| **3** | **SEL+ (category: binary)** | beta=-0.01/year  [-0.33,0.32] | p=0.974 |
| **4** | **PRL count at baseline (n)** | beta=0.04/year  [-0.01,0.08] | p=0.080 |
| **5** | **PRL volume* at baseline (ml)** | beta=0.02/year  [-0.001,0.05] | p=0.084 |
| **6** | **PRL+ (category: binary)** | beta=0.15/year  [0.01; 0.30] | **p=0.044** |
| **7** | **SEL+PRL+ (category: binary)** | beta=0.16/year  [0.01; 0.31] | **p=0.032** |
| **8** | **SEL– (category: binary)** | beta=0.01/year  [-0.32; 0.33] | p=0.974 |
| **9** | **definite SEL count (n)** | beta= 0.01  [-0.002; 0.02] | p=0.109 |
|  | **PRL+ (category: binary)** | beta=0.10  [-0.05; 0.26] | p=0.193 |
| **10** | **definite SEL volumes* (ml)** | beta=0.01  [-0.001; 0.01] | p=0.109 |
|  | **PRL+ (category: binary)** | beta=0.11  [-0.05; 0.26] | p=0.181 |
| **11** | **PRL+ (category: binary)** | 0.03  [-0.33; 0.39] | p=0.867 |
|  | **SEL+PRL+ (category: binary)** | 0.14  [-0.23; 0.50] | p=0.461 |

*refers to the log-transformed volumes of the definite SEL category and the PRL category

The table shows the interaction terms between time and the independent variables, MRI measures or patient categories, while the clinical score (EDSS) was the dependent variable. Whenever the interaction term is significant, we assume that there is a significant association between the MRI measure/patient category and the change in the clinical variable over time.

The following lines explain one by one all the models using the identification of the first column of the table:

**Model 1**

Dependent variable = EDSS

Explanatory variables = SEL count (n), time (in years)

Interaction term: time x SELs, Age (at baseline), Gender(male/female), Disease Duration, Total lesion volume, Treatment at end of study (yes/no), Number of relapses during observation

**Model 2**

Dependent variable = EDSS

Explanatory variables = SEL volume (ml), time (in years)

Interaction term: time x SELs, Age (at baseline), Gender(male/female), Disease Duration, Total lesion volume, Treatment at end of study (yes/no), Number of relapses during observation

**Model 3**

Dependent variable = EDSS

Explanatory variables = SEL+ (or ≥1 SEL) category (binary: yes/no)

Interaction term: time x SEL+, Age (at baseline), Gender(male/female), Disease Duration, Total lesion volume, Treatment at end of study (yes/no), Number of relapses during observation

**Model 4**

Dependent variable = EDSS

Explanatory variables = PRL count (n), time (in years)

Interaction term: time x PRL count, Age (at baseline), Gender(male/female), Disease Duration, Total lesion volume, Treatment at end of study (yes/no), Number of relapses during observation

**Model 5**

Dependent variable = EDSS

Explanatory variables = PRL volume (ml), time (in years)

Interaction term: time x PRL volume, Age (at baseline), Gender(male/female), Disease Duration, Total lesion volume, Treatment at end of study (yes/no), Number of relapses during observation

**Model 6**

Dependent variable = EDSS

Explanatory variables = PRL+ (or ≥1 PRL) category (binary: yes/no)

Interaction term: time x PRL+, Age (at baseline), Gender(male/female), Disease Duration, Total lesion volume, Treatment at end of study (yes/no), Number of relapses during observation

**Model 7**

Dependent variable = EDSS

Explanatory variables = SEL+PRL+ (or ≥1 SEL & ≥1 PRL) category (binary: yes/no)

Interaction term: time x SEL+PRL+, Age (at baseline), Gender(male/female), Disease Duration, Total lesion volume, Treatment at end of study (yes/no), Number of relapses during observation

**Model 8**

Dependent variable = EDSS

Explanatory variables = SEL- (or 0 SEL) category (binary: yes/no)

Interaction term: time x SEL-, Age (at baseline), Gender(male/female), Disease Duration, Total lesion volume, Treatment at end of study (yes/no), Number of relapses during observation

**Model 9**

Dependent variable = EDSS

Explanatory variables = SEL count (n), PRL+ category (binary: yes/no)

Interaction term: time x SEL count

Interaction term: time x PRL+, Age (at baseline), Gender (male/female), Disease Duration, Total lesion volume, Treatment at end of study (yes/no), Number of relapses during observation

**Model 10**

Dependent variable = EDSS

Explanatory variables = SEL volume (ml), PRL+ category (binary: yes/no)

Interaction term: time x SEL volume

Interaction term: time x PRL+, Age (at baseline), Gender(male/female), Disease Duration, Total lesion volume, Treatment at end of study (yes/no), Number of relapses during observation

**Model 11**

Dependent variable = EDSS

Explanatory variables = SEL+PRL+ category (binary: yes/no), PRL+ category (binary: yes/no)

Interaction term: time x SEL+PRL+, time x PRL+, Age (at baseline), Gender(male/female), Disease Duration, Total lesion volume, Treatment at end of study (yes/no), Number of relapses during observation
